# The side effects of the pandemic on all-cause postoperative mortality in a COVID reference Hospital in Brazil: a before and after cohort study with 15156 patients

**DOI:** 10.1016/j.bjane.2025.844600

**Published:** 2025-02-22

**Authors:** Luciana C. Stefani, Brasil Silva Neto, Débora Roberta de Avila Dornelles, Mariana Brandão, Marcio Rahel Guimarães, Pedro Knijnik, Jeruza N. Neyeloff, Stela M.J. Castro, Paulo Corrêa da Silva Neto, Gilberto Braulio

**Affiliations:** a Universidade Federal do Rio Grande do Sul (UFRGS), Faculdade de Medicina, Programa de Pós-Graduação em Ciências Médicas, Porto Alegre, RS, Brazil; b Hospital de Clínicas de Porto Alegre, Serviço de Anestesia e Medicina Perioperatória, Porto Alegre, RS, Brazil; c Hospital de Clínicas de Porto Alegre, Serviço de Urologia, Porto Alegre, RS, Brazil; d Universidade Federal do Rio Grande do Sul (UFRGS), Faculdade de Medicina, Porto Alegre, RS, Brazil; e Universidade Federal do Rio Grande do Sul (UFRGS), Departamento de Medicina Social, Porto Alegre, RS, Brazil; f Universidade Federal do Rio Grande do Sul (UFRGS), Departamento de Estatística, Porto Alegre, RS, Brazil

**Keywords:** Coronavirus, COVID-19, Pandemic, Postoperative mortality, Surgical death, Surgical risk

## Abstract

**Background:**

Before the pandemic, healthcare systems in Low-Middle Income Countries (LMIC) experienced a limited capacity to treat postoperative complications. It is uncertain whether the interference of the Coronavirus (COVID-19) pandemic on surgical systems has increased postoperative mortality.

**Methods:**

This before and after cohort study aimed to assess the pandemic's impact on in-hospital postoperative mortality in a university COVID-19 reference hospital in southern Brazil. Data from patients who underwent surgery before (January 2018 to December 2019) the pandemic were compared to data from patients who underwent surgery during the pandemic (February to December 2020). The primary outcome was in-hospital mortality. We developed Poisson regression models to examine the mortality risk of being operated on during the COVID-19 pandemic.

**Results:**

We assessed 15156 surgical patients, 12207 of whom underwent surgery before the pandemic and 2949 during the first year of the pandemic. Mortality rates were 2.5% (309/12207) in the pre-pandemic versus 7.2% (212/2949) in the pandemic. Of these, 25.8% (32/124) of patients with COVID-19 and 6.4% (80/2816) of patients without COVID-19 died. The proportion of urgent surgeries and ASA-PS III was higher in the pandemic group. After adjusting for mortality-related variables, the Relative Risk (RR) associated with undergoing surgery during the pandemic was 1.51 (95% CI 1.27 to 1.79). We excluded COVID-19-positive to perform a sensitivity analysis that confirmed the increased risk of undergoing surgery during the pandemic RR = 1.50 (95% CI 1.27 to 1.78).

**Conclusion:**

The substantial number of additional deaths, even amongst those without COVID-19 infection, suggests the pandemic disrupted the surgical service in an LMIC context. Fragile surgical systems may suffer more significant adverse impacts from external stressors such as a pandemic, and urging measures are needed to increase their performance and resilience.

**Keypoints**

• The COVID-19 Pandemic disrupted surgical health care in Brazil. Postoperative 30-day in-hospital mortality increased for non-infected patients during the pandemic. To maintain quality, surgical health systems in low-middle-income countries must be more resilient to external stressors.

Alt-text: Unlabelled box

## Introduction

When health systems face an outbreak of pandemic diseases and are overwhelmed, patients struggle to access essential care. These conditions increase mortality by direct and indirect factors, such as neglected preventable and treatable conditions.^1^ The COVID-19 pandemic displaced many health system priorities with a negative impact on patient safety and management of diseases, especially surgical conditions.^2^ In Brazil, official statistics attribute more than 600000 deaths directly to COVID-19 since the onset of the pandemic. Although Brazil is considered an upper-middle-income country, multiple layers of geographical and socioeconomic inequalities exist in healthcare access and health outcomes.^3^ The initial spread of COVID-19 and the resultant deaths were mainly driven by patterns of socioeconomic vulnerability, with the socioeconomically deprived North and Northeast regions particularly affected. Hospital human resources, measured by the numbers of Intensive Care Unit (ICU) personnel and infrastructure per capita, were approximately twice as high in the Southern region compared to the Northern region. These inequalities led to higher COVID-19 death rates in Brazil's most socioeconomically vulnerable states.^4^

Early in the pandemic, the government declared the Hospital de Clínicas de Porto Alegre a regional reference hospital responsible for treating critically ill patients with COVID-19 in the south of Brazil. Despite doubling the number of ICU beds available, COVID-19 admissions eventually surpassed the operational capacity of the institution. At this point, treating critically ill patients outside the ICU became necessary in adapted clinical wards and previously surgical units. This adapted system provided the means to overcome the healthcare crisis.^5^ Still, assessing the consequences of these adaptative changes to the health system, including the surgical system, is paramount.

At the peak of the outbreak of COVID-19, healthcare managers decided to postpone elective and low-intermediate severity surgeries. Also, due to the reorganization of the health system and measures imposed to control the spread of the disease, referral to specialized care was severely restricted. Even oncological procedures had to be delayed due to the lack of available places or hospital resources, creating a substantial backlog in treating these conditions.

Even before the pandemic, the surgical system in LMIC (low-middle income countries) already experienced a limited capacity to rescue and scale up care, leading to many missed opportunities to prevent deaths due to gradual postoperative physiological deterioration in hospital wards.^6^,^7^ Although our institution developed a successful pathway to treat high-risk patients, it was necessary to interrupt this line of care to assist COVID-19 patients.^8^

Studies have proven that COVID-19 infection is an independent risk factor for perioperative mortality, with rates as high as 20%.^9^,^10^ However, it remains unclear whether the pandemic itself or hospital assistance has played a role in postoperative outcomes in patients without COVID-19 undergoing surgery during the pandemic.

To assess the independent effect of the pandemic on healthcare assistance and surgical outcomes in a public reference hospital in southern Brazil, we conducted a retrospective cohort study of surgical patients that underwent surgery during the pandemic. We compared it with a cohort of patients who had surgery before the pandemic. We hypothesized that the unprecedented demands due to the pandemic negatively impacted the surgical outcome, leading to increased postoperative death rates amongst patients with and without COVID-19.

## Methods

The study protocol was approved by the Brazilian National Research Ethics Committee (CAAE 31063220400005327, Chairperson Têmis Maria Félix) with a waiver for written informed consent. However, researchers signed a confidentiality agreement to access the institution's database. We collected only routine, anonymized data from the electronic medical record to assess changes in care processes and clinical outcomes from patients operated on during and before the pandemic. We report this study following the STROBE guideline.

### Site

The Hospital de Clínicas de Porto Alegre (HCPA) is an 800-bed, public tertiary care university teaching hospital in southern Brazil. Early in the pandemic, the Brazilian federal government appointed the HCPA as a regional reference hospital for treating critically ill patients with COVID-19. The hospital increased its service capacity by more than 200%. It underwent several adaptations to allow for the growing number of patients with COVID-19 admitted. ICU beds increased from 62 before the pandemic to 256 during peak months. Eventually, the number of patients requiring care surpassed the operational capacity and critically ill patients with acute respiratory failure underwent treatment outside ICUs, dedicating the COVID-19 ward to patients requiring advanced respiratory support. An infrastructure increase and healthcare workers’ hiring occurred at unprecedented speed. Most medical consultations went online, and the delivery of surgical services was adjusted according to the pandemic waves. Nonetheless, COVID-19 mortality in our hospital was 23.0% in August 2021.^5^

### Data source and studied population

This is an observational single-center cohort using retrospective data from patients who underwent surgical treatment before the pandemic, from January 2018 to December 2019, and during the pandemic, from March to December 2020. For both cohorts, we included patients older than 16 with a planned overnight stay, except patients who underwent diagnostic or ambulatory procedures. We followed the patients until discharge or for 30 days, whichever came first.

We collected the data from our institutional electronic health records and restricted the analysis to complete cases only. We collected data on clinical and demographic characteristics, COVID-19 status, surgical characteristics and the nature of the procedure.

After March 2020, under institutional guidelines, patients were generally screened for COVID-19 with Reverse Transcriptase-Polymerase Chain Reaction (RT-PCR) assays via nasopharyngeal swabs before surgery. When symptoms were present, the institutional protocol recommended a computed chest tomography or chest X-Ray to investigate lung abnormalities. Institutional guidelines recommended postponing elective surgeries if COVID-19 was confirmed or suspected.

The exposure of interest was the effect of the pandemic on surgical care. The primary outcome was in-hospital postoperative mortality, censored 30 days after surgery for patients remaining in the hospital beyond this point.

### Variables definition and derivation

We used variables derived from the Ex-care risk model^11^,^12^ to adjust the mortality risk to the main exposure variable: undergoing surgery during the pandemic. The Ex-Care model was developed and validated with data from the same institution and includes four variables associated with postoperative mortality: age, American Society of Anesthesiology Physical Status (ASA-PS) classification, the severity of the procedure, and the nature of surgery. The model results in a probability of 30-day in-hospital mortality categorized into four classes: class I, < 2%; class II, 2%‒4.99%; class III, 5%‒9.99%; class IV, ≥ 10%. The authors considered classes III and IV to represent high-risk surgical patients. A non-proprietary smartphone application of the risk model is available on Android and iOS platforms.^13^ We defined age as recorded on the first surgical procedure admission. We classified the severity of the surgery as major or non-major according to a previously validated risk model.^11^ We considered only the major procedure when multiple codes were associated with surgery on a single day. We classified surgical specialties: upper/lower gastrointestinal and hepatobiliary as general; breast, head & neck, gynecological, and plastic surgery as other; and we kept vascular, orthopedic, urologic, thoracic, and neurosurgery as independent categories.

### Statistical analysis and variables adjustments

There are two cohorts of interest: before the pandemic (control group) and during the pandemic. We collected the same variables from the electronic medical records for both groups. We show clinical data as number (proportions), Mean ± Standard Deviation or Median [Interquartile Range]. We used the Ex-care risk model^11^ to adjust the individual risk of death, and according to the risk classes, we evaluated the occurrence of the primary outcome. We used a *Z* test with Bonferroni correction to compare mortality in each separate risk class before and after the pandemic.

We examined the independent association between in-hospital 30-day mortality for the two groups (usual care and during the pandemic) using Poisson regression with robust error variance for the primary outcome analysis. To control for potential confounding factors, we adjusted the model for a group of variables based on a conceptual framework describing the relationship between risk factors.^14^ Variables associated with the patient's risk (ASA-PS, age) and to surgical procedure entered in the model (urgent vs. elective, major vs. non-major and surgical specialty). We also built a separate model considering the EX-Care risk model classes plus the presence of active COVID-19 infection and surgical specialty. To check the independent effect of the pandemic on general surgical mortality, we conducted a sensitivity analysis in which we excluded surgical patients with a positive COVID-19 test. We show the models’ Relative Risk (RR) and 95% Confidence Intervals (95% CI).

This cohort study includes all patients who underwent surgery in the Hospital de Clínicas de Porto Alegre during the study period. We did not anticipate the sample size, but we calculated the *post-hoc* statistical power of the result after data analysis with all the predictors included in the model. The significance level for all statistical analyses is 5%. We performed the analysis using the Statistical Analysis System (SAS Studio® 9.4) and R 3.5.1.

## Results

**Figure 1:**
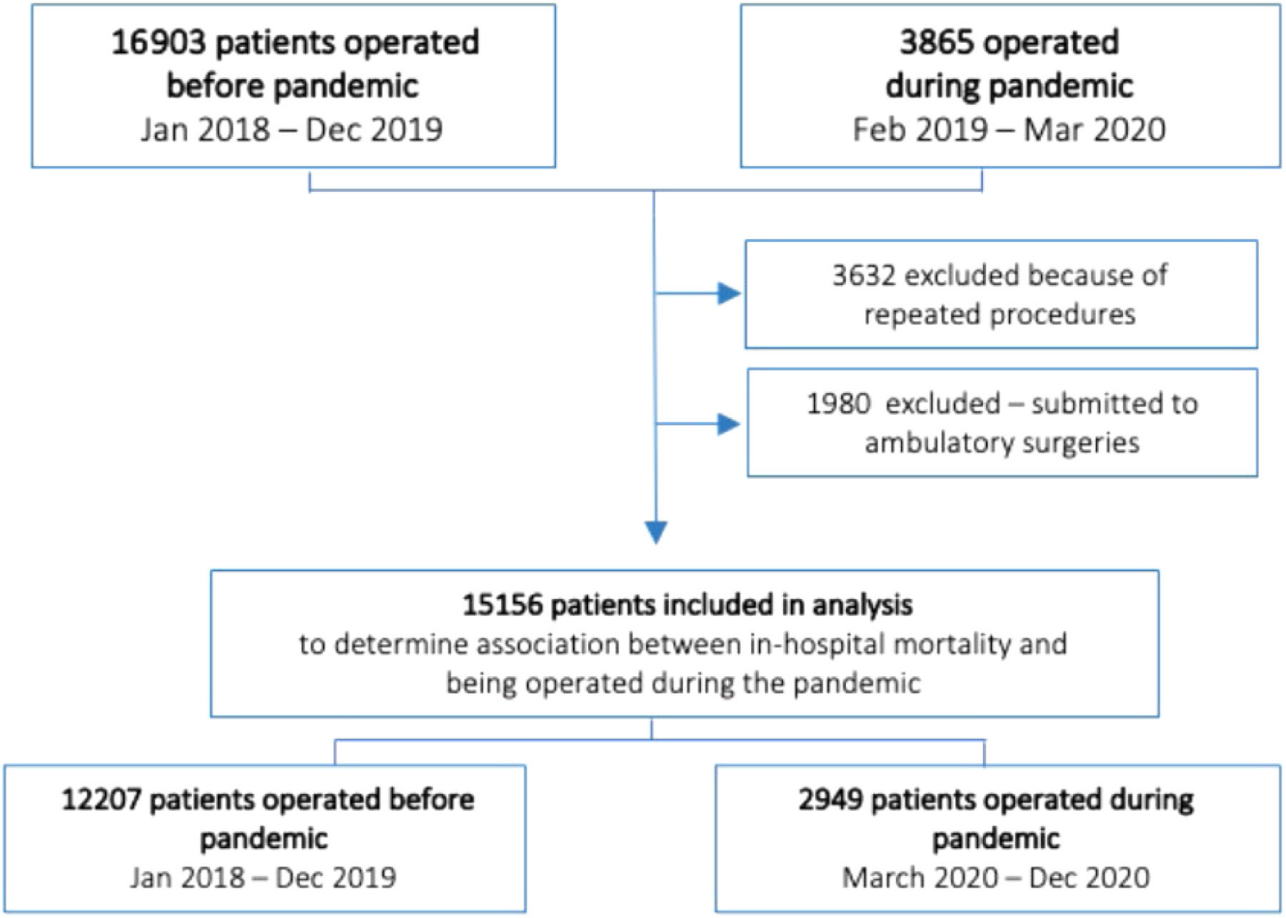
Patient flow diagram showing cases included in both cohorts.

There was a total of 20768 surgical procedures in the Hospital de Clínicas de Porto Alegre between January 2018 to December 2020. After excluding repeated or outpatient procedures, we analyzed 15156 single surgical patients, 12207 before the pandemic, and 2949 during the pandemic year. Figure 1 describes the patient's inclusion in the study. Urgent surgeries occurred in 2473 (20.3%) patients of the control group versus 1042 (35.4%) patients in the pandemic group. Before the pandemic, major surgery occurred in 3058 (25%) patients and ASA III or higher composed 3677 (30.1%) of those cases. However, during the pandemic, major surgery occurred in 995 (33.7%) patients, and 1325 (44.9%) were ASA III or higher.

We observed an increase in the proportion of high-risk patients operated on during the pandemic. Ex-Care predicted high-risk patients (Predicted 30-day mortality between 5.0% and 9.9%) were 584 (4.8%) in the control group and 250 (8.5%) in the pandemic group. In comparison, very high-risk patients (Predicted 30-day mortality ≥ 10%) were 755 (6.2%) in the control group and 353 (12%) in the pandemic group. Among the 2949 adult patients who underwent surgery during the pandemic, 124 (4.2%) had COVID-19. We show the baseline characteristics of the study population in Table 1.

**Table 1:** Clinical and surgical characteristics of patients operated before or during the pandemic. Values are mean ± SD or number (proportion).

	Before the pandemic		During the Pandemic	
	Year 2018 (n = 6109)	Year 2019 (n = 6098)	COVID negative (n = 2825)	COVID positive (n = 124)
Age in years	53.63 ± 16.8	54.61 ± 16.5	54.36 ± 17.29	55.5 ± 15.46
ASA-PS				
I	1096 (17.9%)	836 (13.7%)	285 (10.1%)	4 (3.2%)
II	3247 (53.2%)	3351 (55%)	1309 (46.5%)	17 (13.7%)
III	1528 (25%)	1658 (27.2%)	993 (35.3%)	69 (55.6%)
IV	213 (3.5%)	220 (3.6%)	194 (6.9%)	31 (25%)
V	25 (0.4%)	33 (0.5%)	35 (1.2%)	3 (2.4%)
Risk of death (Ex-Care model)^a^				
Predicted mortality < 2%	4684 (76.7%)	4606 (75.5%)	1781 (63.2%)	48 (38.7%)
Predicted mortality 2%‒54.99%	748 (12.2%)	830 (13.6%)	490 (17.4%)	18 (14.5%)
Predicted mortality 5.0%‒9.9%	296 (4.8%)	288 (4.7%)	223 (7.9%)	27 (21.8%)
Predicted mortality ≥10%	381 (6.2%)	374 (6.1%)	322 (11.4%)	31 (25%)
Surgical specialties				
General Surgery	2241 (36.7%)	2176 (35.7%)	1076 (38.2%)	24 (19.4%)
Vascular	650 (10.6%)	706 (11.6%)	410 (14.6%)	17 (13.7%)
Thoracic	165 (2.7%)	131 (2.1%)	113 (4%)	38 (30.6%)
Orthopedic	519 (8.5%)	576(9.4%)	226 (8%)	6 (4.8%)
Urology	1015 (16.6%)	1079 (17.7%)	463 (16.4%)	13 (10.5%)
Neurosurgery	204 (3.3%)	170 (2.8%)	159 (5.6%)	8 (6.5%)
Others^b^	1315 (21.5%)	1260 (20.7%)	369 (13.1%)	18 (14.5%)
Major Surgery	1542 (25.2%)	1516 (24.9%)	957 (34%)	38 (30.6%)
Urgent surgery	1294 (21.2%)	1179 (19.3%)	996 (35.4%)	46 (37.1%)
In-hospital death	145 /6109 (2.4%)	164 /6098 (2.7%)	180 /2816 (6.4%)	32 /124 (25.8%)

a Ex-Care risk model was determined using calculator available online (Br J Anaesth. 2021;126:525-32).

b Breast, Head & Neck, Gynecological, and Plastic Surgeries.

### Patient outcomes

**Figure 2:**
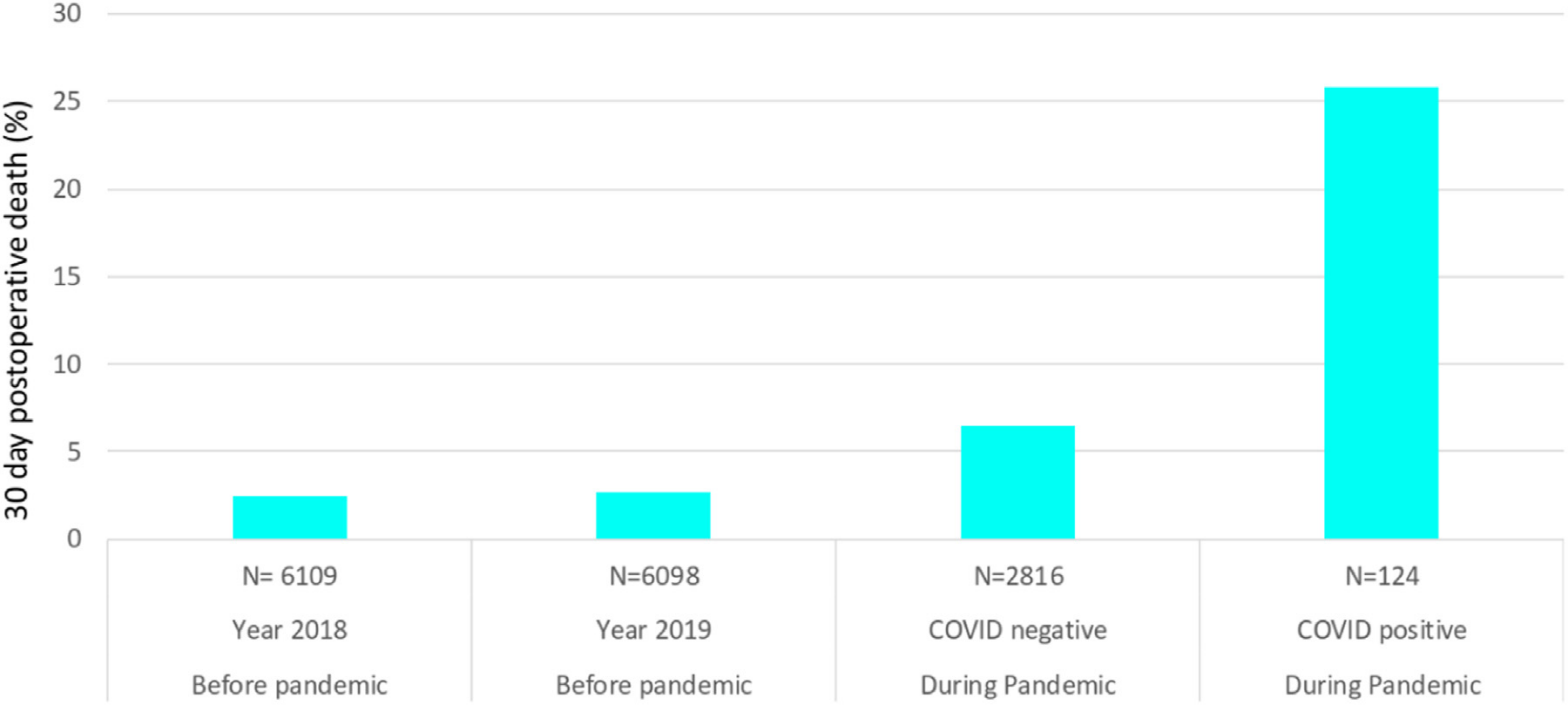
Postoperative mortality in the pre-pandemic cohort and the pandemic cohort among patients with and without COVID.

Overall, 521/15156 (3.43%) patients who underwent a surgical procedure died during the study period. We observed a significant increase in 30-day postoperative deaths during the pandemic. The mortality rate was 2.5% (309/12207) in the pre-pandemic control group versus 7.2% (212/2949) in the pandemic cohort. Mortality was 25.8% (32/124) among patients with COVID-19 and 6.4% (180/2816) among patients without COVID-19 during the same period (Figure 2).

### Mortality according to risk classes

**Figure 3:**
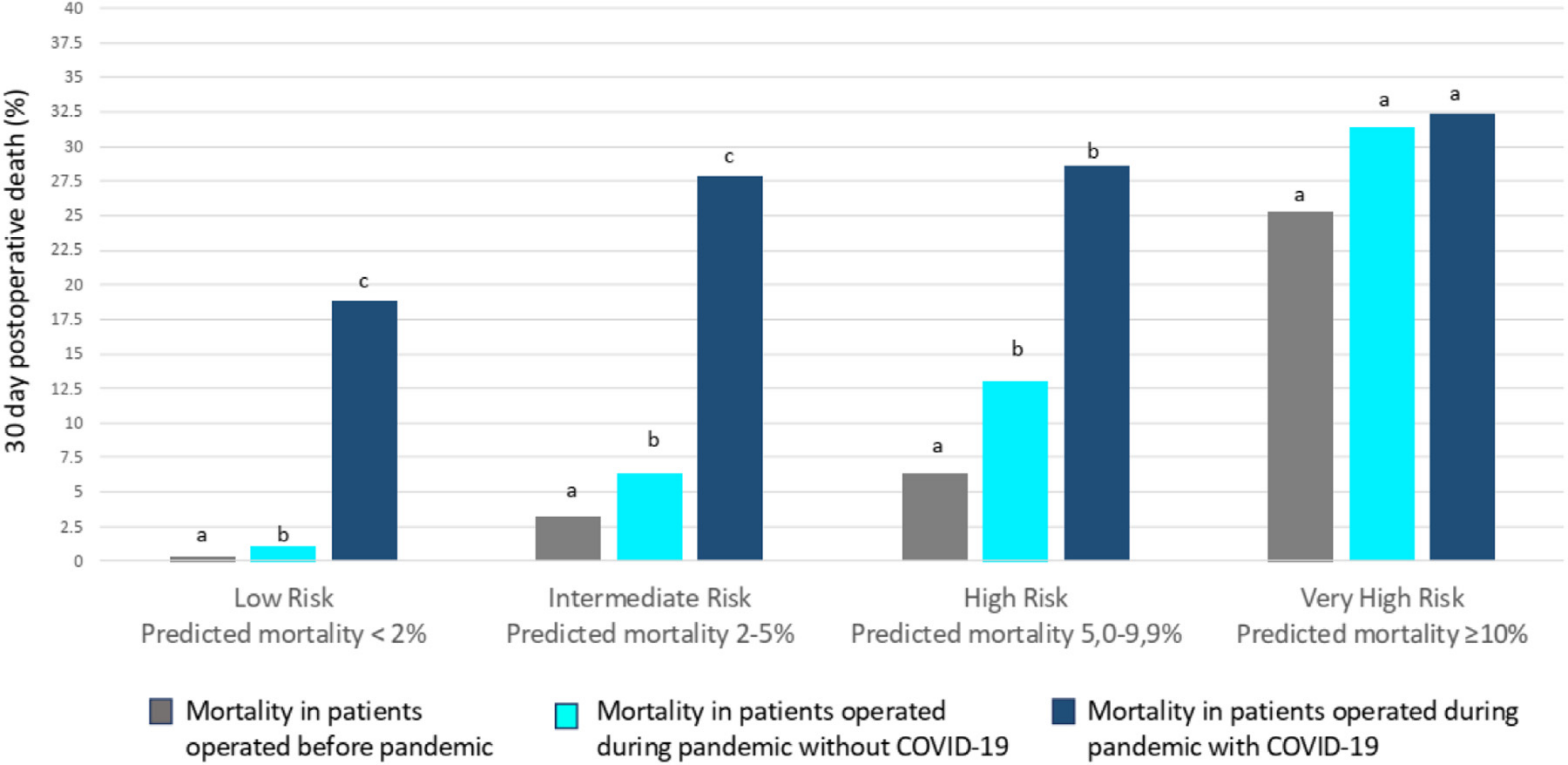
30-day mortality according to risk class based on Ex-Care risk model in patients operated before the pandemic (grey), during the pandemic without COVID-19 (light blue) and during the pandemic with COVID-19 (dark-blue). The percentage of deaths was compared separately for each risk class. Note that different letters reflect the significant between-group differences in each class (*Z* test with Bonferroni correction).

We identified an increased death rate during the pandemic in patients without COVID-19. We found a 100% increase in the death rate in the intermediate (from 3.2% to 6.3%) and high-risk classes (from 6.3% to 13%). Patients in the very high-risk category (≥ 10% probability of death) had a non-significant increase in death rate (from 25% to 31.4%) (Figure 3).

### Risk of death of being operated during the pandemic

The unadjusted Relative Risk (RR) of death in the pandemic group was 2.84 (95% CI 2.40 to 3.37). After adjustment for variables comprising the baseline risk of mortality in several risk models (age, ASA-PS, severity and urgency of procedure),^15^, ^16^, ^17^ plus the presence of COVID-19 infection and surgical specialties, the effect of undergoing surgery during the pandemic (effect group) remained significant, with an adjusted RR of 1.51 (95% CI 1.27 to 1.79). We also found independent associations between mortality and COVID-19 infection RR = 1.93 (95% CI 1.28 to 2.90), age RR = 1.02 (95% CI 1.02 to 1.03), ASA-PS RR = 3.42 (95% CI 3.09 to 3.78), urgent procedures RR = 2.46 (95% CI 2.01 to 3.01) and thoracic specialty RR = 2.54 (95% CI 1.91 to 3.37). Table 2 shows the detailed model. To evaluate the independent effect of the pandemic on mortality, we ran another model using the Ex-care risk categories for risk adjustment (Supplemental Digital Content Table S1). In this confirmatory analysis, we obtained an RR for postoperative mortality of 1.52 (95% CI 1.27 to 1.84) for being operated on during the pandemic. We calculated a *post-hoc* power of 99.6% for this result, including 15147 subjects (2949 in the pandemic group), adjusted for five covariates (those used in the final model shown in Table 3), considering 5% significance.

**Table 2:** Unadjusted and adjusted association between pandemic exposure and in-hospital mortality in 15147 patients according to clinical and surgical risk factors.

	Relative Risk (95% CI)	p-value
**Unadjusted model (n = 15147)**		
Pandemic exposition	2.84 (2.40 to 3,37)	<0.001
**Adjusted model (n = 15147)**		
**Pandemic Exposition**	1.51 (1.27 to 1.79)	<0.001
Covid-Positive	1.93 (1.28 to 2.90)	<0.01
Age	1.02 (1.02 to 1.03)	<0.001
ASA-PS	3.42 (3.09 to 3.78)	<0.01
Non-elective vs. elective	2.46 (2.01 to 3.01)	<0.001
Major surgery vs. non-major	1.17 (0.98 to 1.39)	0.079
**Surgical specialties**		
General Surgery	Ref (0)	
Vascular	0.77 (0.62 to 0.96)	0.025
Thoracic	2.54 (1.91 to 3.37)	<0.01
Orthopedic	0.72 (0.46 to 1.11)	0.145
Urology	0.60 (0.43 to 0.84)	0.003
Neurosurgery	1.03 (0.79 to 1.35)	0.800
Others^a^	0.44 (0.28 to 0.7)	<0.01

CI, Confidence Interval; ASA-PS, American Society of Anesthesiology Physical Status.

a Breast, Head & Neck, Gynecological, and Plastic Surgeries.

**Table 3:** Unadjusted and adjusted association between pandemic group and in-hospital mortality in 15023 patients according to clinical and surgical risk factors excluding COVID patients.

	Relative Risk (95% CI)	p-value
**Unadjusted model (n = 15023)**		
Pandemic group	2.52 (2.11 to 3.021)	<0.001
**Adjusted model (n = 15023)**		
**Pandemic Group**	1.50 (1.26 to 1.78)	<0.001
Age	1.02 (1.02 to 1.03)	<0.001
ASA-PS	3.53 (3.17 to 3.92)	<0.001
Non-elective vs. elective	2.47 (2.0 to 3.06)	<0.001
Major surgery vs. non-major	1.19 (0.99 to 1.43)	0.057
**Surgical specialties**		
General Surgery	Ref (0)	
Vascular	0.78 (0.62 to 0.97)	0.032
Thoracic	2.53 (1.87 to 3.42)	<0.01
Orthopedic	0.70 (0.43 to 1.11)	0.134
Urology	0.59 (0.42 to 0.83)	0.003
Neurosurgery	1.02 (0.78 to 1.34)	0.857
Others^a^	0.36 (0.21 to 0.60)	<0.01

CI, Confidence Interval; ASA-PS, American Society of Anesthesiology Physical Status.

a Breast, Head & Neck, Gynecological, and Plastic Surgeries.

### Sensitivity analysis of postoperative in-hospital mortality without COVID-positive patients

We conducted a sensitivity analysis in which we excluded the COVID-19-positive patients. Repeating the Poisson regression model, the pandemic effect remained significant, with an adjusted RR of 1.50 for postoperative mortality (95% CI 1.26 to 1.78) (Table 3).

## Discussion

The COVID pandemic displaced many health system priorities, negatively impacting the treatment of many diseases.^18^ The main finding of this study was that postoperative mortality substantially increased among patients, with or without COVID-19, undergoing surgery during the pandemic at a university COVID-19 reference hospital in Brazil. For non-COVID-19 patients operated on during the pandemic peak, the risk of dying was 1.5 times that of the pre-pandemic period. A multivariable model adjusted for clinical and surgical risk factors demonstrated the independent effect of the pandemic on postoperative mortality. As far as we know, this is the first study that showed an independent pandemic effect on the mortality of surgical patients not infected with COVID-19 in a LIMC context.

Chronic under-resourcing in health systems has hampered the response to the pandemic throughout Latin America, which has 18.4% of the cumulative confirmed deaths from COVID-19.^18^ Several pandemic-related factors, such as disruption to the drug and equipment supply chains, interrupted therapies, staff shortages and reassignment, and delayed detection of new cases led to excess morbidity and mortality linked to other diseases.^19^ Some studies evaluated the indirect impact of the COVID-19 pandemic on delayed cancer screening,^20^ and on cardiac complications.^21^,^22^ For surgical frameworks that struggle to deliver better care in low-middle-income countries, the stressor of a pandemic, which disrupts the health system, may cause even greater adverse events than that of organized and well-funded ones. This situation is of great concern in Brazil,^23^ where health-system preparedness was decentralized and varied greatly through regions. The side effects of the pandemic on health assistance were expected but needed to be thoroughly examined.

This pragmatic study cannot precisely identify all system failures, but it will help find where the problems related to surgical assistance rest. When comparing our data with the current literature, we found a similar significant increase in mortality rates among surgical patients positive for COVID-19 infection.^10^ A single-institution study in Italy reported a postoperative mortality rate of 19.5% among patients with COVID-19.^9^ In the COVIDSurg international multicenter observational cohort involving 1128 surgical patients who had a pre or postoperative diagnosis of COVID-19, the 30-day mortality was 23.8%.^24^ In another cohort of 140231 patients,^28^ the same research group found an increase in mortality in patients who had surgery at 0–2-weeks, 3–4-weeks and at 5–6-weeks after COVID-19 diagnosis with a respective Odds Ratio (95% CI) of 4.1 (3. To 4.8), 3.9 (2.6 to 5.1) and 3.6 (2.0 to 5.2). This study grounded the current guideline to postpone elective surgeries at least six weeks after COVID-19 infection.^28^

When we look at patients operated on during the pandemic, the recent wide population epidemiological study of NHS surgical patients showed a low risk of in-hospital death (one in 1000) among COVID-19-negative patients undergoing surgery. Measures to prevent nosocomial infection were successfully adopted, including household isolation and dedicated green pathways for COVID-19-negative patients. In our cohort, whilst the mortality rate among COVID-19-positive patients was comparable to other studies (25.8%), the mortality among general patients without COVID-19 was higher, 180/2816 (6.4%), than in any other report on postoperative mortality in large international cohorts from different continents before^25^, ^26^, ^27^ or during the pandemic.^28^ The excess of overall mortality rates compared to the mortality in the previous years occurred in all risk classes. It cannot be attributed exclusively to patients and surgical factors.

Multiple processes underpinned the worst outcomes in surgical care in our institution during the pandemic. Firstly, the institution restricted elective surgical procedures to a minimum, while the hospital's capacity increased by 200%. Also, in the peak months of the pandemic, the surgical schedule contemplated only patients with advanced conditions of diseases or those requiring non-elective surgeries. Surgical patients experienced delays in their treatments and consultations, exacerbating the frailties already present between primary and tertiary care in our Public Health System. Secondly, to assist a four million population region, the hospital rapidly increased the number of COVID-19 dedicated professionals and opened new ICU beds to treat COVID-19 patients. Surgical wards became COVID-19 units, and the displacement of human resources from the Operating Theatre to attend to this new demand occurred. Thirdly, the unprecedented necessity for ICU beds led to the reduced availability of postoperative ICU to high-risk surgical patients, which hampered their assistance. Finally, there was a real threat of disruption to clinical and surgical services. Ward surgical pathways were discontinued, such as the co-management for hip fracture, the path dedicated to high-risk surgical patients, or enhanced recovery programs. The displacement of physiotherapists and clinical rescue teams led to suboptimal postoperative care for these vulnerable groups, especially in rescuing postoperative complications.

Our data suggest that the pandemic disrupted our healthcare system, and we could not deliver surgical care safely during the pandemic. The strengths of this study include the significant number of patients evaluated in both cohorts. Being a single-center study with a tightly matched control population allows us to evaluate the impact of the pandemic on surgical processes. We used a validated risk stratification tool, developed for the Brazilian population, the Ex-Care risk model,^11^ to standardize the risk of death for individual patients, which made the precise comparison possible.

Our study has some limitations. Firstly, it is vulnerable to bias regarding its cohort design nature. However, the before-and-after cohort design has a pragmatic approach and can capture relevant outcomes using an appropriate comparison group.^29^ Secondly, its results may not reflect the Brazilian or LMIC reality since it is a single-center study in a country with profound inequalities in health assistance. Thirdly, we based our assessment of COVID-19 status on routine pre-operative tests. Finally, we evaluated in-hospital mortality without evaluating long-term mortality, complications, or other clinical and patient-centered outcomes.

In conclusion, we demonstrated that the COVID-19 pandemic disrupted routine surgical services and negatively impacted patient outcomes in a reference hospital in Brazil. The pandemic exacerbated healthcare disparities significantly, leaving a residual impact on patients who experienced delays in curative surgical treatments. We showed that the surgical needs of LMICs still need to be met and that external factors may cause disruption in these fragile systems. Our results will contribute to the urgent need to integrate health information and rethink models to deliver safe and efficient surgical assistance in a strong and resilient system capable of coping with external stressors.

## Conflicts of interest

The authors declare no conflicts of interest.

## Appendix Supplementary materials

Image, application 1

## Footnotes

Supplementary material associated with this article can be found in the online version at doi:10.1016/j.bjane.2025.844600.
