# Supplementary material for: The side effects of the pandemic on all-cause postoperative mortality in a COVID reference Hospital in Brazil: a before and after cohort study with 15156 patients
Source: Braz J Anesthesiol. 2025 Feb 22;75(2):844600. doi: 10.1016/j.bjane.2025.844600 (PMC11914783; doi:10.1016/j.bjane.2025.844600)
Supplement: Supplementary file 1 [file mmc1.docx]

**BJAN-D-25-00010_ Supplementary Material**

**Supplemental Digital Content Table S1** Unadjusted and adjusted association between pandemic cohort and in-hospital mortality in 15147 patients adjusted according to Ex-care risk classes and surgical specialties.

|  | **RR (95% IC)** | **p-value** |
| --- | --- | --- |
| **Unadjusted model (n = 15147)** |  |  |
| Pandemic group | 2.84 (2.40‒3.37) | <0.001 |
| **Adjusted model (n = 15147)** |  |  |
| **Pandemic Group** | 1.52 (1.27‒1.84) | <0.001 |
| **Covid-Positive** | 1.76 (1.19‒2.60) | <0.004 |
| **Ex-Care Risk model^a^** |  |  |
| Ref Predicted mortality < 2% | Ref (0) |  |
| Predicted mortality ≥ 10% | 39.14 (29.14‒52.57) | <0.001 |
| Predicted mortality 5.0%‒9.9% | 13.64 (9.58‒19.43) | <0.001 |
| Predicted mortality 2%‒5% | 7.26 (5.12‒10.24) | <0.001 |
| **Surgical specialties** |  |  |
| General Surgery | Ref (0) |  |
| Vascular | 0.68 (0.54‒0.87) | 0.02 |
| Thoracic | 2.03 (1.51‒2.72) | <0.01 |
| Orthopaedic | 0.62 (0.39‒0.68) | 0.042 |
| Urology | 0.48 (0.33‒0.68) | <0.01 |
| Neurosurgery | 1.75 (0.89‒1.54) | 0.246 |
| Others** | 0.43 (0.26‒0.7) | 0.01 |

^a^ Ex-Care risk model was determined using calculator available online https://excarebr.app.

Gutierrez CS, Passos SC, Castro SMJ, et al. Few and feasible preoperative variables can identify high-risk surgical patients: derivation and validation of the Ex-Care risk model. Br J Anaesth. 2021;126:525-32.

Passos SC, de Jezus Castro SM, Stahlschmidt A, et al. Development and validation of the Ex-Care BR model: a multicentre initiative for identifying Brazilian surgical patients at risk of 30-day in-hospital mortality. Br J Anaesth. 2024;133:125-34.
